# A deep learning model for classifying human facial expressions from infrared thermal images

**DOI:** 10.1038/s41598-021-99998-z

**Published:** 2021-10-19

**Authors:** Ankan Bhattacharyya, Somnath Chatterjee, Shibaprasad Sen, Aleksandr Sinitca, Dmitrii Kaplun, Ram Sarkar

**Affiliations:** 1 University of Kentucky, Lexington, KY USA; 2 Computer Science and Engineering Department, Future Institute of Engineering and Management, Kolkata, India; 3 Computer Science and Technology Department, University of Engineering and Management, Kolkata, India; 4 Department of Automation and Control Processes, Saint Petersburg Electrotechnical University “LETI”, Saint Petersburg, Russia; 5 Department of Computer Science and Engineering, Jadavpur University, Kolkata, India

**Keywords:** Expression systems, Imaging

## Abstract

The analysis of human facial expressions from the thermal images captured by the Infrared Thermal Imaging (IRTI) cameras has recently gained importance compared to images captured by the standard cameras using light having a wavelength in the visible spectrum. It is because infrared cameras work well in low-light conditions and also infrared spectrum captures thermal distribution that is very useful for building systems like Robot interaction systems, quantifying the cognitive responses from facial expressions, disease control, etc. In this paper, a deep learning model called *IRFacExNet* (**I**nfra**R**ed **Fac**ial **Ex**pression **Net**work) has been proposed for facial expression recognition (FER) from infrared images. It utilizes two building blocks namely Residual unit and Transformation unit which extract dominant features from the input images specific to the expressions. The extracted features help to detect the emotion of the subjects in consideration accurately. The Snapshot ensemble technique is adopted with a Cosine annealing learning rate scheduler to improve the overall performance. The performance of the proposed model has been evaluated on a publicly available dataset, namely *IRDatabase* developed by RWTH Aachen University. The facial expressions present in the dataset are Fear, Anger, Contempt, Disgust, Happy, Neutral, Sad, and Surprise. The proposed model produces 88.43% recognition accuracy, better than some state-of-the-art methods considered here for comparison. Our model provides a robust framework for the detection of accurate expression in the absence of visible light.

## Introduction

According to Mehrabian et al.^1^, 55% of the messages are conveyed through facial expression, 7% through linguistic language (verbal), and 38% through paralanguage like intonation (vocal) for the communication of human beings feelings. Owing to the wide range of applications, Facial Expression Recognition (FER) is considered an important research topic in the research fraternity. The Facial Action Coding System (FACS), introduced by Ekman and Friesen^2^ and Ekman et al.^3^ in the field of Psychology, refers to a set of muscle movements known as Action Units (AUs) that correspond to the specific emotions. These emotions can be decoded manually by observing the muscle movements with the naked eyes from a video taken with visible light. This kind of facial expression analysis by using FACS-AU depends on human observation through naked eyes and has no mathematical evidence of the expressions related to the facial expressions. Hence, FACS-AU is considered as a qualitative approach and has many ambiguities, like neutral face, and smiling face may be identified as the same facial expression.

Recently, researchers are showing their interest in developing systems for FER using machine learning (ML) and deep learning (DL) approaches, which is not only paving the way for the development of robust FER systems but also discovering new parameters used for FER. Generally, the images used for the classification of facial expressions are taken by the cameras using visible light, because these cameras are easily available not only as handheld cameras but also in attachment with portable devices like phones and tablets, which are cheap. Though there are many works available on the recognition of facial expressions from images captured by cameras that use ordinary light in the visible spectrum^4–8^, the classification of expressions becomes a tough job because of daily conditions like a shadow, reflection, and darkness (or low-light). Moreover, other objects like background pictures, scenery, and many other things exist along with the face. Therefore, it becomes an overhead to extract the face from the image to analyze the facial expressions. Working with thermal images helps to solve these issues as it considers the thermal distribution in facial muscles and provides better facial expression classification.

Due to its various advantages, thermal imaging is used in different real-life applications. A few of such applications are mentioned in this section. Authors in^9^ have shown that thermal imaging can play an important role to facilitate Human-Robot Interaction. Goulart et al.^10^ have provided a new perspective to the application of IR imaging which can be utilized to understand physiological signals to make them significant in social interactions. The suggested method also helps to recognize the cognitive processes by involuntary expressions. Clay-Warner et al.^11^ have highlighted how IR imaging can be used to quantify the amount of arousal by detecting the expressions. It also helps the robot to identify the emotion of humans during an interaction. Owing to the recent outbreak of the coronavirus (COVID-19) virus, thermal cameras are used to detect the skin temperature of passengers, which in turn helps to detect fevers, in the airport and other such places, helping in disease control.

In the present study, we have proposed *IRFacExNet*, a DL-based model for the classification of facial expressions from thermal images. *IRFacExNet* extracts features with the help of residual and transformation units. The generated features are passed through a fully connected layer, which is finally used for the classification of facial expressions into eight different emotions namely, Fear, Anger, Contempt, Disgust, Happy, Neutral, Sad, and Surprise. The concept of snapshot ensemble has also been used in the present work to achieve better recognition accuracy in comparison to the existing works on the IRDatabase.

The key contributions of the current work are as follows:

1. Proposed *IRFacExNet* can classify the facial expressions from the thermal images more accurately.
2. The applied snapshot ensemble technique (which is based on cosine annealing) can enhance the prediction capability.
3. The model outperforms many existing FER methods on the IRDatabase.

The rest of the paper is organized as follows: “Literature survey” section discusses the recent works related to FER on both thermal and normal images. “Data analysis” section provides a brief discussion on the IRDatabase^12^ used in the current experimentation. “Proposed methodology” section elaborates the proposed methodology used for the FER with the details of the architecture, snapshot ensembling, and training process. “Results and discussion” section describes the obtained outcomes by the proposed model with exhaustive analysis. Finally, “Additional experiments” section concludes the work with the pros and cons of the proposed architecture and also highlights the future scope of the work.

## Literature survey

As stated earlier, research on building FER systems from images captured by cameras using visible light has been popular due to the easy availability and low cost of such cameras. Hammal et al.^13^ classified facial expressions from videos by fusing facial deformation using a rule-based decision with the help of the framework known as the transferable belief model (TBM), which considers simple distance coefficients based on simple facial features like eyes, mouth, and eyebrows, etc. Ojo et al.^14^ classified two facial expressions, fear, and sadness using a local binary pattern histogram on two databases. One database is the Japanese female facial expression (JAFFE) database and the other one is the Cohn cade database. Kyperountas et al.^15^ developed a multi-step two-class classification problem for facial expressions and reported results on the JAFFE and MMI databases. During each step of the process, among many two-class classifiers, the best classifier is identified. This helped the authors to come up with a better FER system. Ali et al.^16^ proposed a two-step method for classifying facial expressions. First, the facial features were extracted using a histogram of oriented gradients (HOG), and then a sparse representation classifier (SRC) was used to classify the facial expressions. Bartlett et al.^17^ used a combination of Adaboost and Support vector Machine (SVM) to recognize facial expressions to be used in the human-robot interaction assessment. With the advent of deep learning architecture, the need for manually extracting features is no longer important, as techniques like convolution and max-pooling can be used as a part of a model, stacked in layers, to extract features and feeding the features into classifiers. Authors in^18^ have shown a DL-based method that exploits the spectral correlation and the spatial context to extract more relevant features used in their experiment. Rodriguez et al.^19^ proposed a DL-based architecture called Deep Pain, to detect pain automatically by classifying facial expressions. Here, instead of manually extracting the facial features, the face is directly fed into a Convolution Neural Network (CNN) linked to Long Short-Term Memory (LSTM) to remove long-term dependencies. From the classified facial expressions, the model can predict the type of pain a patient experiences. One of the simplest processes is to detect FACS-AU by passing facial features to models like the Hidden Markov Model (HMM) to decode the emotions. Similar work has been performed by Lien et al.^20^ by extracting facial features using three modules and feeding them into a discriminant classifier or HMM to classify them into FACS-AUs.

As stated earlier, due to other objects and conditions, it becomes too difficult to classify the facial expressions as there is an overhead for face detection at different poses and also due to the qualitative nature of FACS-AU, there are ambiguities in classifying facial expressions as these vary from the person to person depending on the angle of viewing. So, to make only the face part visible, thermal images are used. Thermal images allow only the skin of the human to be exposed when the emissivity value is set to near 1^21^. Moreover, in thermal images, the thermal distribution in facial muscles is detected. This fact allows better facial expression classification and leaves no room for ambiguity as it is not dependent on external factors like human viewing through naked eyes and inconvenient lighting conditions. In contrast, the fact that IRTI cameras are less affordable by the common people due to cost, database consists of thermal images of facial expressions is not in abundance. As a result, only a few research works have been performed in this area, and hence there is a greater room for research in this domain. Jiang et al.^22^ used geometric characteristics to recognize facial expressions. Yoshitomi et al.^23^ used a 2-dimensional method to detect temperature distribution in the face and passed the features into a neural network to classify the facial expressions. Authors have dealt with four expressions (Neutral, Happy, Surprise, and Sad) and achieved 90% recognition accuracy. Bijalwan et al.^24^ proposed a principal component analysis (PCA) and distance classifier to classify facial expressions. Shen et al.^25^ used a FER system using video data. Primarily, horizontal and vertical temperature differences are used to extract sequential features, and then they have used an FS-technique to find the most relevant features for the classification purpose. Eventually, the selected features are passed to the Adaboost algorithm using the K nearest neighbors (KNN) classifier to classify the facial expressions. The database used for the experiment is Natural Visible and Infrared facial Expression (USTC-NVIE) database, and the authors have achieved 75.3% and 76.7% classification accuracies between high versus low arousal and valence. Goulart et al.^26^ used visual and thermal image processing for recognition of facial emotions in child-robot interaction. Authors have concentrated on the recognition of five emotions (disgust, fear, happiness, sadness, and surprise) and shown mean accuracy of 85.75%. Khan et al.^27^ developed a FER system based on infrared measurement of facial skin temperature variations. Authors have obtained 66.28% cross-validation accuracy and 56% person-independent classification accuracy, respectively. Prabhakaran et al. in^28^ proposed a model for emotion detection by predicting facial expressions using ResNet152 on NVIE dataset. In this experiment, authors have shown that Residual networks are easier to optimize and produce good recognition accuracy by increasing the depth. Authors in^29^ have shown that facial expression can widely be used in biometric and security applications. They have utilized the efficiency of deep learning methods for disguise invariant face recognition by incorporating a noise-based data augmentation method. The authors have reported 98.19% recognition accuracy on the DFW dataset. Bodavarapu et al. built a method called FERConvNet_HDM for the recognition of facial expressions applied on FER2013 and LRFE datasets^30^. The proposed mechanism combines Gaussian, Bilateral and Non-local means denoising Filter that helps to increase the performance of the CNN in turn by achieving 85% and 95% accuracies in FER2013 and LRFE datasets, respectively. Reddy et al. in^31^ proposed a technique that combines hand-crafted and deep learning features to recognize facial expressions on the AffectNet database. The authors have extracted hand-crafted features from facial landmark points and XceptionNet was used for the extraction of deep features. The authors have achieved 54%, 58%, and 59% accuracies using their proposed model on three sets of data distributions (Imbalanced set, Down-sampled set, and Up-sampled set). Looking into the various applications of facial expression recognition and the volume of works presented in the literature, it is clear that there is a great scope to work in this field.

## Data analysis

**Figure 1:**
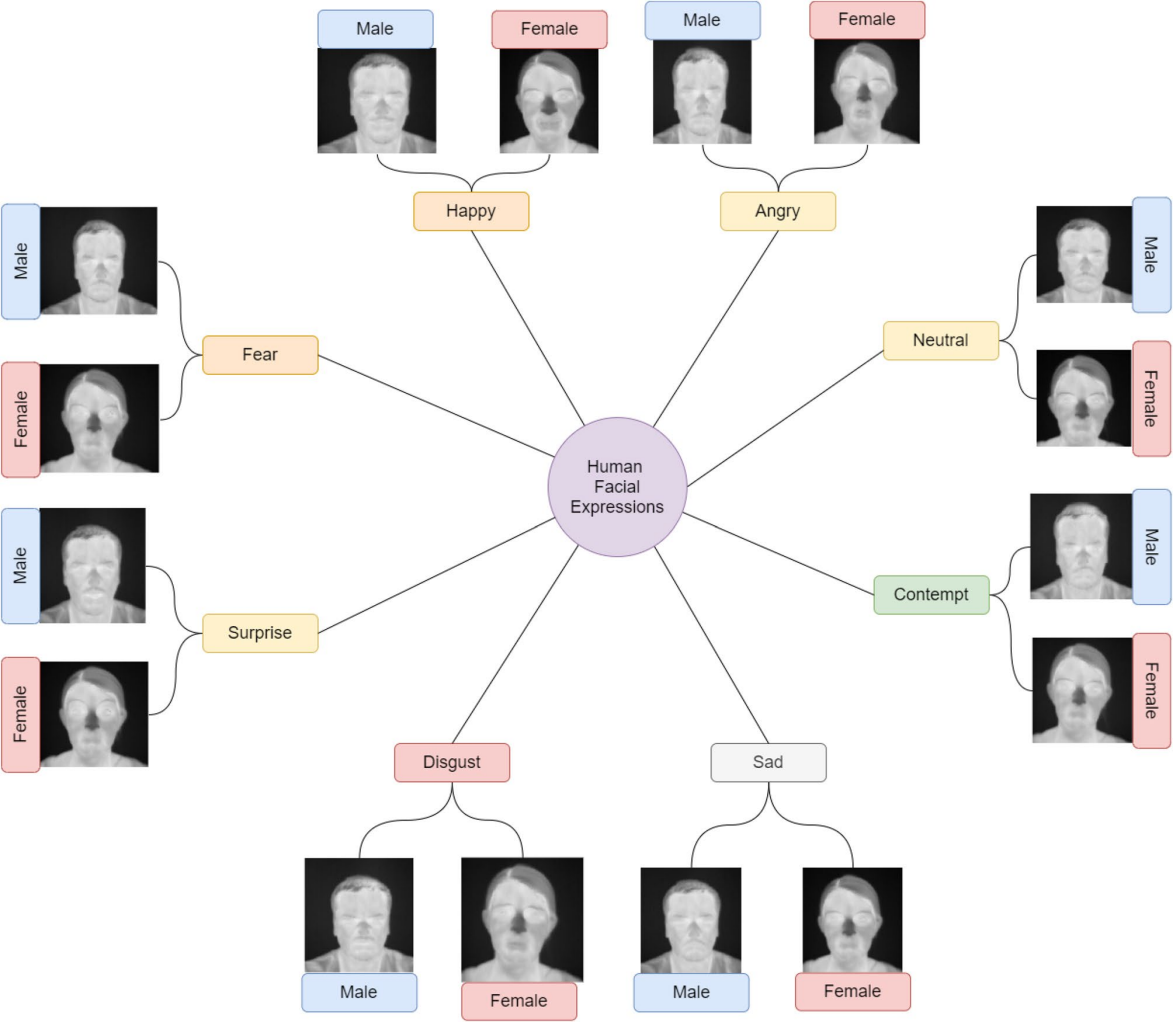
Sample images representing all eight expressions, happiness, sadness, surprise, fear, disgust, anger, contempt and neutral for males and females. Image is developed using Draw.io^32^.

For the current work, we have used the database published by Kopaczka et al.^12^. The images of this database were recorded using an Infratec HD820 high-resolution thermal infrared camera with a 1024 768 pixel-sized microbolometer sensor with a thermal resolution of 0.03 K at 30 $^{\circ }$C and equipped with a 30-mm f/1.0 prime lens. Subjects were filmed while sitting at a distance of 0.9 m from the camera, resulting in a spatial resolution of the face of approximately 0.5 mm per pixel. A thermally neutral backdrop was used for the recordings to minimize background variation. To build the database, video recordings of the subjects acquired with a frame rate of 30 frames/s were manually screened and images were extracted. The database contains a total of 1782 sample images with eight classes of expressions^12^ namely, Fear, Anger, Contempt, Disgust, Happy, Neutral, Sad, and Surprise. The number of sample images belonging to each class has been shown in Table 1. A few sample images from the considered database have been shown in Fig. 1.

**Table 1:** Counts of each facial expression in the *IRDatabase*.

Expression	Number of samples
Angry	231
Contempt	189
Disgust	225
Fear	210
Happy	234
Neutral	231
Sad	231
Surprised	231
**Total**	**1782**

Bold value indicates total number of samples used in the experiment.

In the proposed work, as a pre-processing step, we have converted the input images into grayscale images and reshaped them into a uniform size of 200 $\times$ 200 pixels before feeding to the network.

## Proposed methodology

The present study deals with Human Facial Expression Recognition (HFER) using thermal infrared images acquired from the database mentioned in^12^. Several studies exist in the literature for the processing of facial expressions when the image is taken in the visible spectrum. However, these systems are not directly applicable for infrared facial processing, rather we can utilize the ideas after a certain amount of tailoring to obtain competent results for the infrared images^33^. It is to be noted that there exist some limitations when dealing with infrared images for HFER that include lack of colors for the distinction of facial features, obscure skin folds, and wrinkles that are formed on the forehead or cheeks indicating specific expressions like happiness or anger. The hotspot is a relatively common problem with infrared imaging which is a blurry bright spot in the center of the image. The presence of a hotspot makes the expression detection task more challenging.

Recently, it has been observed that the rise of CNNs greatly influenced the building of efficient HFER systems. It has proved its extraordinary success when working with RGB images. Here we try to exploit the strengths of deep CNN architecture for feature extraction which in turn will help to recognize different facial expressions more accurately. The proposed *IRFacExNet* architecture is specially designed to work with infrared images. This network efficiently extracts useful information from the input images for the detection of facial expressions. To make the predictions more accurate an efficient ensemble strategy (known as Snapshot Ensemble)^34^ has been adopted. It is to be noted that this ensemble technique helps to achieve better performance with no computational overhead^35^. During the training process, any CNN model gets converged to several local minima along the optimization path. In this approach, we save the corresponding models at regular intervals (known as snapshots). In the later stage, these snapshots are combined by an aggregation rule to create an ensemble classifier. The detailed architecture of the proposed model and the snapshot ensemble technique are explained elaborately in the following subsection.

### Proposed architecture

Instead of using traditional convolution units, the proposed *IRFacExNet* utilizes depth-wise convolutions, where each input channel is convolved by each filter channel separately. This is followed by a point-wise convolution of a 1x1 window to merge the channel-wise extracted features. The standard convolution does channel-wise and spatial convolution in one step. On the other hand, depth-wise convolution used in the proposed model splits this into two steps, which helps to lower the number of trainable parameters and thus reduces the chances of overfitting. These depth-wise convolutions have been used with varying dilation rates for a global perspective and to capture the spatial and cross-channel correlations of the inputs.

**Figure 2:**
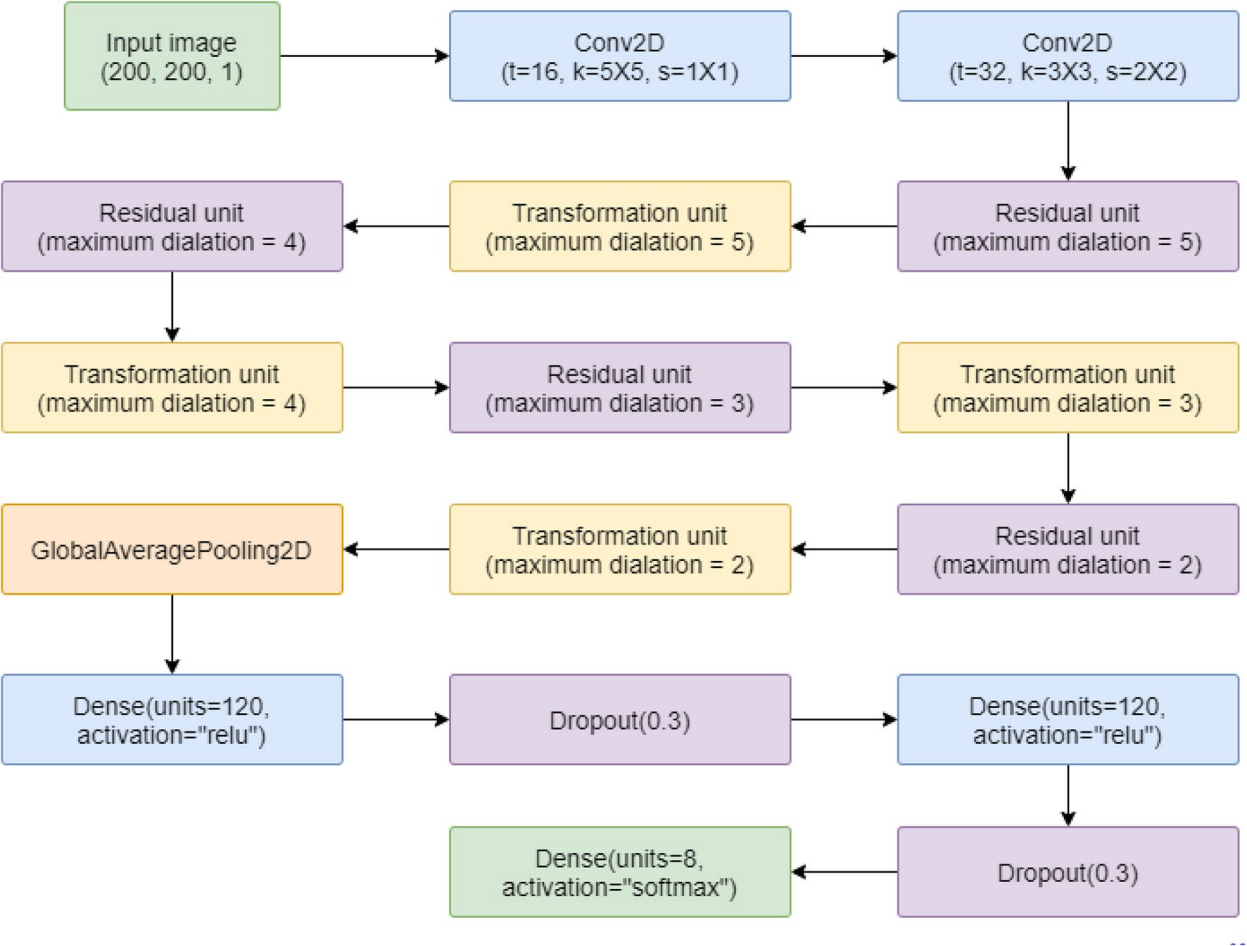
A schematic diagram of the proposed *IRFacExNet* model used for HFER. Image is developed using Draw.io^32^.

The proposed *IRFacExNet* model shown in Fig. 2 is divided into two structural units, namely Residual Unit, and Transformation Unit. Dilated convolution is the primary innovation incorporated in these units to extract variegated features from the entire image. The working procedure of dilated convolutions, Residual Unit, and Transformation Unit has been mentioned below.

**Figure 3:**
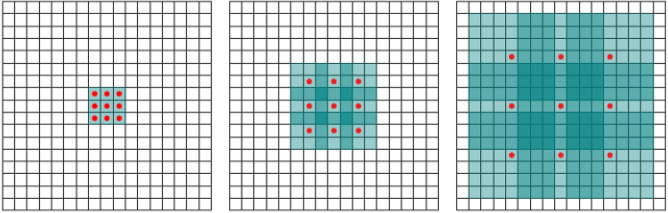
Schematic representation of dilated convolutions with dilation rate l, l = 1 (left), l = 2 (Middle), l = 4 (Right).

For accurate classification of facial expressions, it is important to extract distinctive features that are unique to an expression while covering the complete image from different perspectives. Dilated convolutions broaden the receptive field and help to bring diversity to the feature maps with no increase in trainable parameters^34^. Figure 3 shows the working procedure of the dilated convolutions.

**Figure 4:**
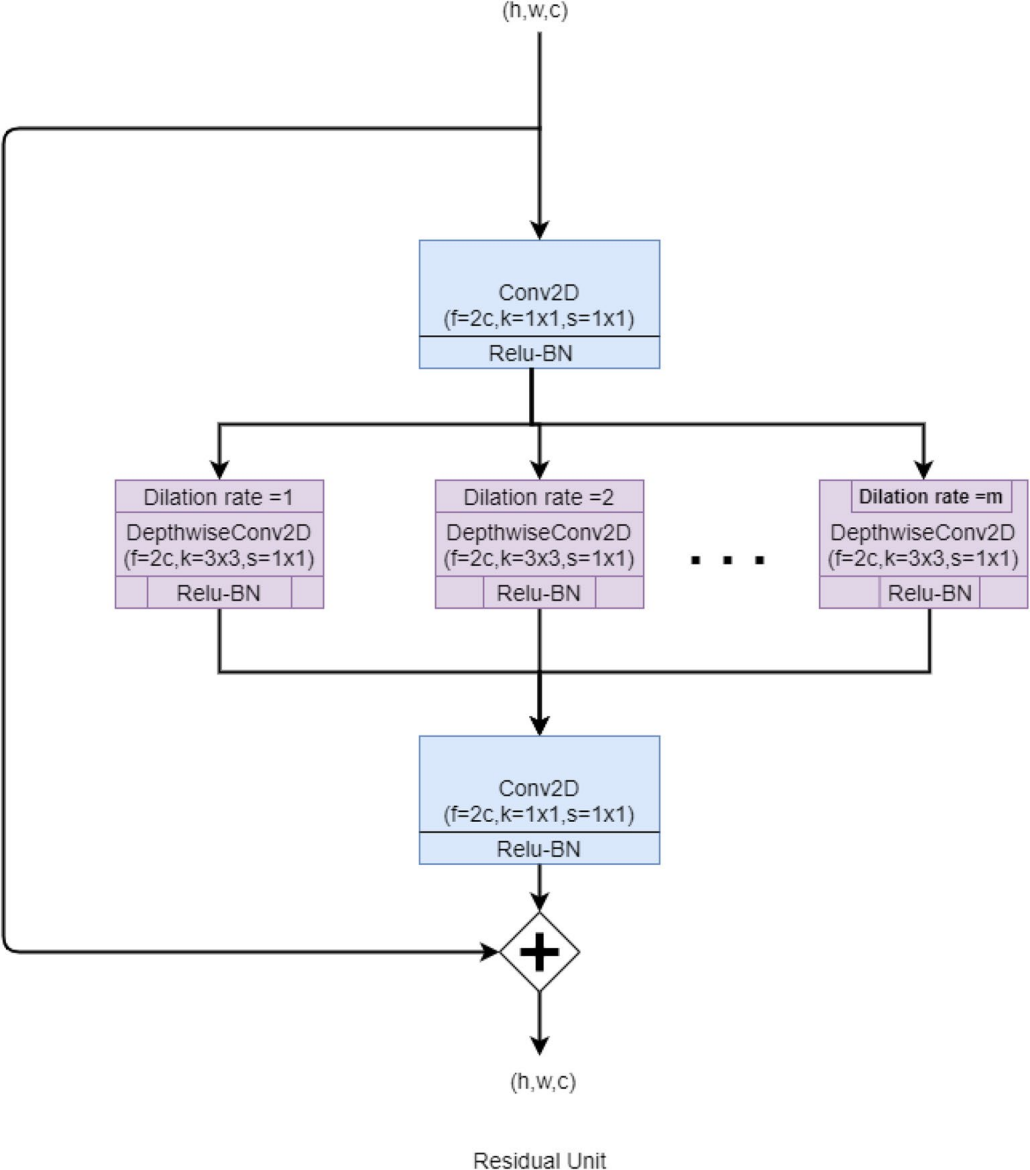
The basic block diagram of the Residual Unit of the proposed *IRFacExNet* model. Image is developed using Draw.io^32^.

In the proposed structural units, an input image first goes through a point-wise convolution which projects the inter-channel information into a broader space. It is then propagated through several depth-wise convolutions with varying dilation rates from 1 to m which broaden the receptive field for feature extraction and help to find the localized features as well as the features that are distributed over the wide area. Thus it helps to find spatial and cross-channel features from different perspectives. Finally, these varied features are merged using the point-wise convolutions. In the Residual Unit, the output of the mapping scheme is merged with the input features itself known as residual learning proposed by He et al.^36^. If the input is termed as U and the residual mapping function as R(), then the output can be defined as F: U [U + R(U)]. This allows us to build a deeper network without overfitting and helps the dominant features to propagate deeper into the network without much distortion. It also avoids vanishing gradient problems and the skip connection technique helps to build a much deeper network that is easier to train. After all the internal processing inside the residual block, the output shape is maintained equal to the input shape. The basic block diagram of the Residual Unit has been shown in Fig. 4.

**Figure 5:**
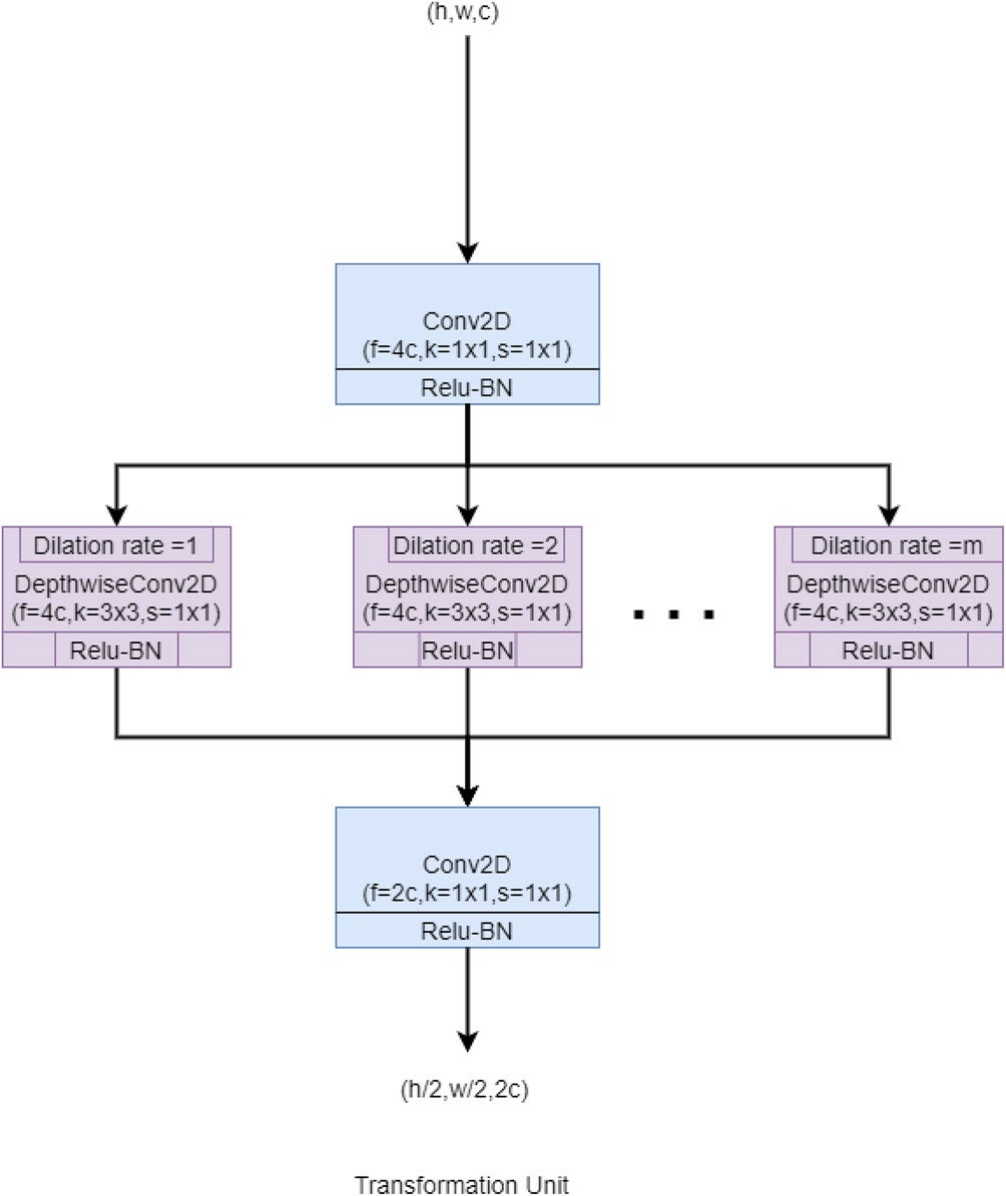
The basic block diagram of the Transformation Unit of the proposed *IRFacExNet* model. Image is developed using Draw.io^32^.

The Transformation Unit can be considered as a substitute for the pooling layers. Here, we have tried to avoid the greedy technique of Max-pooling because it usually causes the loss of diffused features and positional information. The Transformation Unit has been designed similarly to the Residual Unit i.e., point-wise–depth-wise–point-wise convolutions. Here, the first point-wise convolution extends the number of channels four times its input to complement the depth-wise convolutions. Next, the spatial dimensions are halved using the stridden depth-wise convolutions. This helps to eliminate unnecessary details and makes it suitable for further operations. The depth of the output feature map is doubled in the final point-wise convolution to increase the filtering operations in later stages. The basic block diagram of the Transformation Unit has been shown in Fig. 5.

In a nutshell, the working strategy of the proposed *IRFacExNet* model is mentioned as follows:

The initial input is processed by some traditional convolutions with larger-sized kernels to produce an adequate number of channels and spatial dimensions. The major part of the network is stacked with residual blocks of depth (d) to produce a deeper network followed by transformation blocks which are introduced to bring dimensional transformations and to improve the generalization performance. The dilation rate (m) depends on the input size of the feature map. For processing larger sized feature maps, a higher dilation rate is preferred as it makes the receptive field wider which helps to extract both generic as well as complex features. Finally, the feature maps are passed through the global average pooling layer followed by some fully connected layers and the classification layer. All the layers are ReLU (Rectified linear unit) activated to bring the non-linearity. Batch-normalization is used to make the convergence faster and to provide a modest generalization effect. It also helps to decrease the internal covariate shift^37^.

### Snapshot ensemble

An ensemble-based classification model has been adopted to leverage the specialties of several classifiers to achieve better prediction. It helps to build a robust model with better generalization performance, higher accuracy, and lower error rate^35^. There exist several methods involving different aggregation rules to aggregate the predictions from different classifiers. Depending on the aggregation rule adopted, the performance of the ensemble model varies. Some commonly used techniques to combine the predictions from the base classifier are Horizontal Voting Ensemble, Weighted Average Ensemble, Stacked generalization, etc. All these methods work pretty well when the base classifiers have learned from the training data in different ways, thus have different generalization errors. However, everything has a cost, and when using traditional ensemble strategies there is an additional computational overhead of training several base learners from scratch with parameter tweaking to achieve the best performance.

As opposed to the usual approach of training different base learners incurring additional computation power and time, Huang et al.^35^ proposed a method to produce many base learners having different generalization errors from a single training run known as Snapshot ensemble. Stochastic Gradient Descent (SGD) and its extensions are widely used for optimizing neural networks. It is capable of avoiding and escaping saddle points and local minima that are considered a great deal. It is advantageous to converge the models to local minima with flat basins. Different models that might have converged to different local minima can have very similar error rates but tend to make different mistakes which is the exact requirement for creating a robust ensemble classification model. With the increase in the number of trainable parameters, the number of possible local minima also increases exponentially. Therefore it is not surprising that two networks having the same architecture with different initialization and mini-batch ordering will converge to different solutions. These models with variegated convergence can be exploited using ensembling in which multiple neural networks can be trained from different initialization which is combined with horizontal voting. Instead of building (M) models from the scratch, SGD optimizer allows to converge to unique local minima M times and saving the corresponding models which are known as snapshots. After saving the snapshot models, the learning rate is increased to bring the optimizer out of the basin and again converging it to other local minima. Note that the model has different initializations when the learning rate is increased again and it reaches a different local minima. Repeating these steps for M times will provide M base learners having different biases. This cyclic nature of learning rate can be achieved by the method known as Cosine Annealing proposed by Loshchilov et al.^38^, in which the learning rate is abruptly raised and then quickly lowered following a cosine function.

### Training process

When experimenting with numerous hyperparameters for training a deep neural network, it is required to check the effect of the tuning and to draw justification from therein about the performance. However, an optimized model might fail to fit the training data and thus cannot extract useful information from the input data. To cope up with this, we have performed the experiment with architecture depth (d) starting from 1 to 4 and assessed the performance of the model. The architecture depth (d) can be understood as the number of Residual Units to be stacked on each other to make the proposed network deep or shallow. We have achieved the best result for d=2 and similar results for others. To reduce the complexity of the model, a depth of 2 units is used for all the residual blocks. Training DCNN with limited data and without overfitting can be considered a very challenging task. To solve this task Dropout and Batch Normalization is generally used throughout the network that helps to decrease the misclassification rate. Grayscale images of (200 $\times$ 200) resolution are feed to the network for training purposes. The details of the database have already been mentioned in “Data analysis” section. The 85% sample images from this database have been used for training the network and the performance is evaluated on the rest 15% of the database.

Firstly, the proposed *IRFacExNet* is trained using Adam optimizer for 100 epochs having a batch size of 16 samples. Then the Adam optimizer is replaced by SGD as it works best with a cyclic learning rate scheduler. An efficient Snapshot ensemble requires an aggressive cyclic learning rate scheduler to converge to different minima for even minor fluctuations in the learning rate. We have utilized Cosine Annealing as the scheduler with the slight modification that varies the learning rate by following a cosine function. Instead of using abrupt restart after reaching the minima^13^, we have applied a gradual rise in the learning rate that allows the optimizer to explore around the local minima and find a better optimization path. Eq. (1) is the mathematical expression of the scheduler.

1 $$\begin{aligned} lr=\alpha _0+\dfrac{\Delta \alpha }{2} \times \left( 1 + \cos \left( \dfrac{\pi \times e}{h}\right) \right) \end{aligned}$$

where ${\alpha }_0$ lower bound of learning rate $(10^{-5})$, ${\Delta }{\alpha }$ the difference between upper bound and lower bound of learning rate $(10^{-5} - 10^{-2})$, *e* current epoch number $(1 - 500)$, *h* the range of half-cycle of the cosine function (100 epochs).

**Figure 6:**
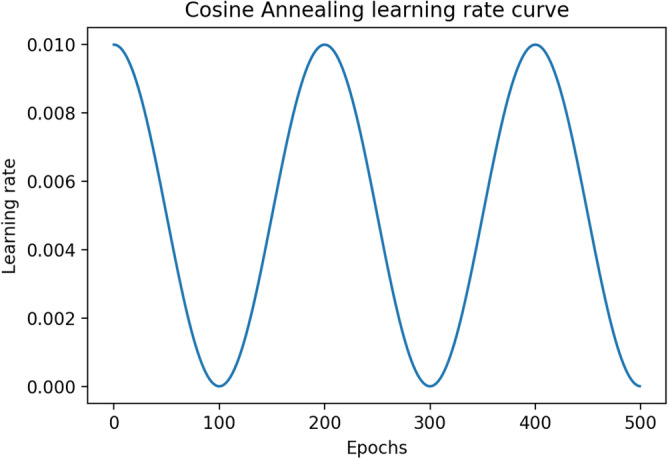
Line plot of the cosine annealing learning rate scheduler used in the training process.

This scheduler considers the parameters like total training epochs, maximum learning rate, minimum learning rate, range of half-cycle, and the epoch number. Figure 6 shows the variability of the learning rate for the training span of 500 epochs. In the present study, we have used the upper bound and lower bound of the learning rate for the scheduler as $10^{-2}$ and $10^{-5}$, respectively. The range of half-cycle has been set to 100 epochs. The snapshot of the model is taken at an interval of 100 epochs and thus producing a total of 5 snapshot models throughout the training process. Due to the cyclic nature of the learning rate, for each snapshot, the model reaches a local minimum having a lower error rate and thus fitting the training data totally (means it has a very high variance). Again, when the model is saved during the rise of the learning rate, it has a comparatively higher error rate and is biased. Such diversity of the model brings a better generalization performance on the unseen data. These developed five snapshot models are combined in all possible combinations for testing purposes and the performance has been estimated on the test data.

Such a type of ensemble model helps to build a robust system and to produce better recognition accuracy. The summary of hyperparameters used for training the model can be found in Table 2.

**Table 2:** Summary of hyperparameters and their values used during the training process.

Hyper-parameter	Value
Type of input images	Grayscale
Resolution of input images	200 $\times$ 200
Learning rate optimizer	SGD optimizer with Cosine annealing scheduler
Learning rate	$10^{-2}$ to $10^{-5}$
Training epochs	500 epochs
Snapshot interval	100 epochs
Batch size during training	16
Residual unit depth (d)	2

## Results and discussion

HFER is considered one of the challenging research problems in the field of computer vision. It is because of the close similarity of human faces among the correlated expressions (like Disgust and Anger). The naive model (without using the snapshot ensemble technique) successfully recognizes the human facial expressions from thermal infrared images with 82.836% classification accuracy, as shown in Table 3. To improve the overall performance of the prediction model, we have employed an ensemble strategy known as Snapshot Ensemble, as stated earlier in “Training process” section. This technique uses the strength of different snapshot models having different biases and helps to predict the final results that improve the performance of the system on unseen data.

**Figure 7:**
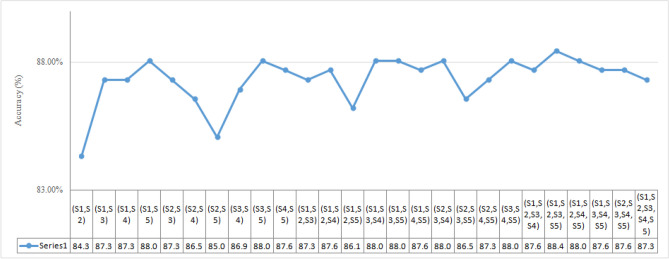
Graph showing the performance of various combinations of Ensemble classifier.

As stated earlier, during the training of the Snapshot ensemble model, an aggressive learning rate scheduler has been used that allows the model to reach a new local minimum each time when there is a drop in the learning rate. When it tries to reach a local minimum, it extracts variegated information from the training data which is dissimilar compared to other local minima and such property is well-suited for the building of an efficient ensemble classifier. We have saved five snapshots, namely S1, S2, S3, S4, and S5 during the complete training process. The snapshots were combined using the voting aggregation rule to generate various ensemble classifiers. For example, snapshot models S1 and S2 are combined to generate (S1, S2), models S1, S2, and S3 are combined as (S1, S2, S3), and so on. The performance of the individual snapshot model (S1 to S5) and all possible combinations are evaluated on the test database. The observed outcomes are reported in Table 3 (Fig. 7).

**Table 3:** Performance of Naïve Model and the snapshot ensemble after combining various snapshots on the test database.

Combinations	Accuracy (%)
Naïve model	82.836
S1	86.16
S2	72.39
S3	87.69
S4	87.69
S5	85.08
(S1, S2)	84.328
(S1, S3)	87.313
(S1, S4)	87.313
(S1, S5)	88.060
(S2, S3)	87.313
(S2, S4)	86.567
(S2, S5)	85.075
(S3, S4)	86.940
(S3, S5)	88.060
(S4, S5)	87.687
(S1, S2, S3)	87.313
(S1, S2, S4)	87.687
(S1, S2, S5)	86.194
(S1, S3, S4)	88.060
(S1, S3, S5)	88.060
(S1, S4, S5)	87.687
(S2, S3, S4)	88.060
(S2, S3, S5)	86.567
(S2, S4, S5)	87.313
(S3, S4, S5)	88.060
(S1, S2, S3, S4)	87.687
**(S1, S2, S3, S5)**	**88.433**
(S1, S2, S4, S5)	88.060
(S1, S3, S4, S5)	87.687
(S2, S3, S4, S5)	87.687
(S1, S2, S3, S4, S5)	87.313

Bold value indicates highest result among all the snapshot conditions.

**Figure 8:**
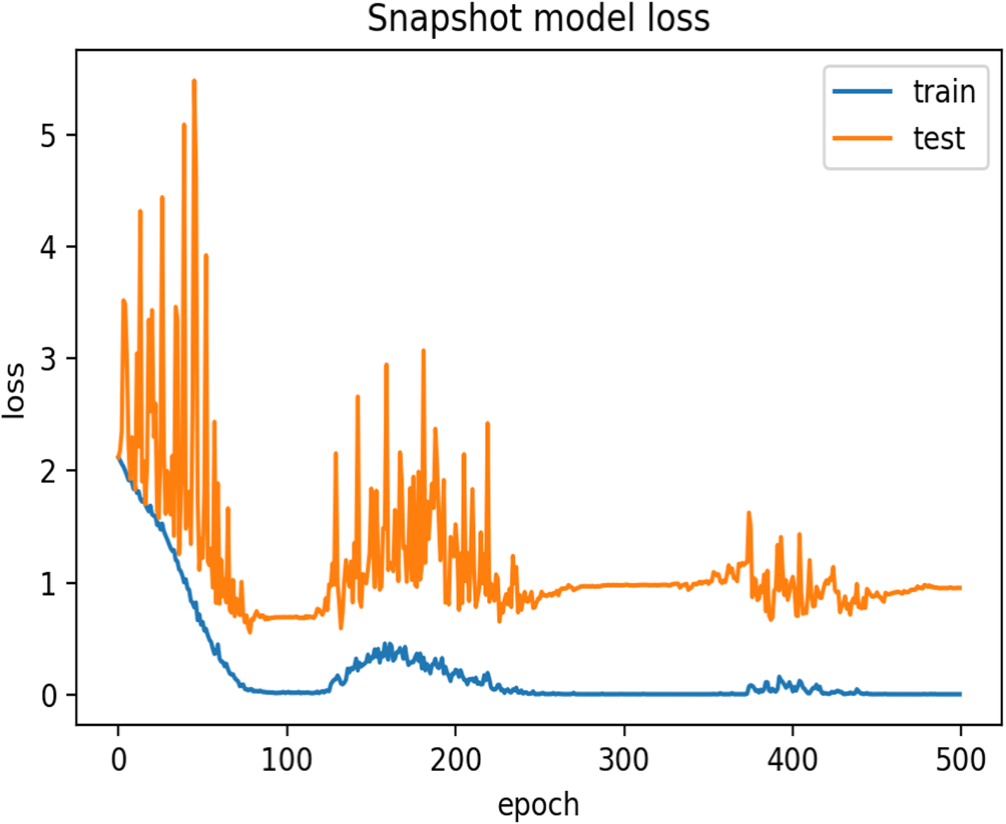
Graph represents loss during the training of IRFacExNet model.

**Figure 9:**
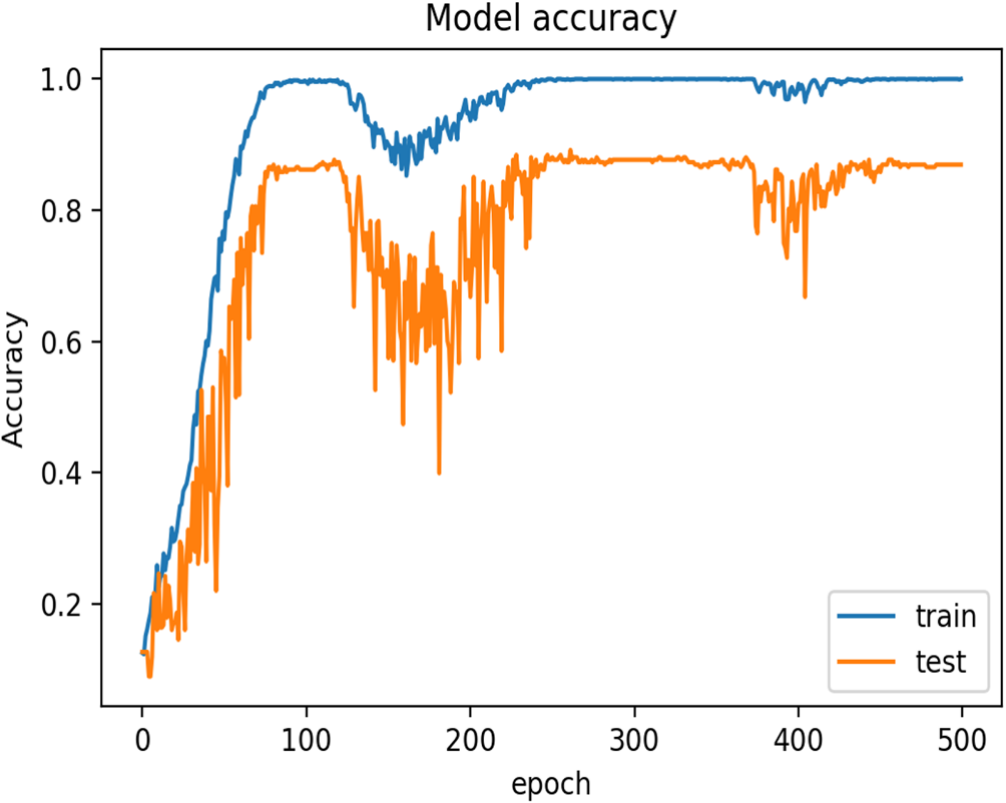
Graph represents the variation of accuracy during the training of IRFacExNet model.

During the training process of the model by using the cosine learning rate scheduler, the first half cycle of the cosine function is completed in 100 epochs (Fig. 6), and the first Snapshot (S1) is generated. During this phase, there is a gradual decrease in the learning rate that helps the optimizer to find local minima. This makes the model perform better for the data similar to the training samples. However, it may not provide good predictions on unfamiliar images. Hence, the learning rate starts increasing gradually and reaches its upper bound, which takes another 100 epochs to produce the second Snapshot S2. This allows the optimizer to explore around the minima and rises along the hill having a gentle slope. The variation of loss during training of the model can be seen in Fig. 8. It helps to decrease the variance of the model and thus improves the overall predictive performance. Therefore, the alternating nature of the learning rate allows the SGD optimizer to bring the models having different properties. The ensemble works best if the base models incorporated in the ensemble have low test errors and do not overlap in the set of examples they fail to classify^35^. Cyclic learning rate scheduler helps to visit several local minima before converging to a final solution. At an interval of 100 epochs, the Snapshots (S1 to S5) are successively considered and are used for the development of the ensemble classifier in the current experimentation. The change in accuracy during the training process can be seen in Fig. 9. All possible combinations of the Snapshot models are assessed on the test data for the performance evaluation. Among all possible combinations of the snapshot models, the best performance has been achieved by the ensemble system when (S1, S2, S3, and S5) are combined to produces 88.433% recognition accuracy as shown in Table 3 and Fig. 7. The possible reason for achieving the best recognition performance when (S1, S2, S3, and S5) are combined is that the considered snapshot models have low test errors and do not overlap in the set of examples.

The naïve model has a recognition accuracy of 82.836% and after adopting the ensemble strategy, the model produced 88.433% recognition accuracy and thereby, increases 5.597% prediction accuracy.

**Figure 10:**
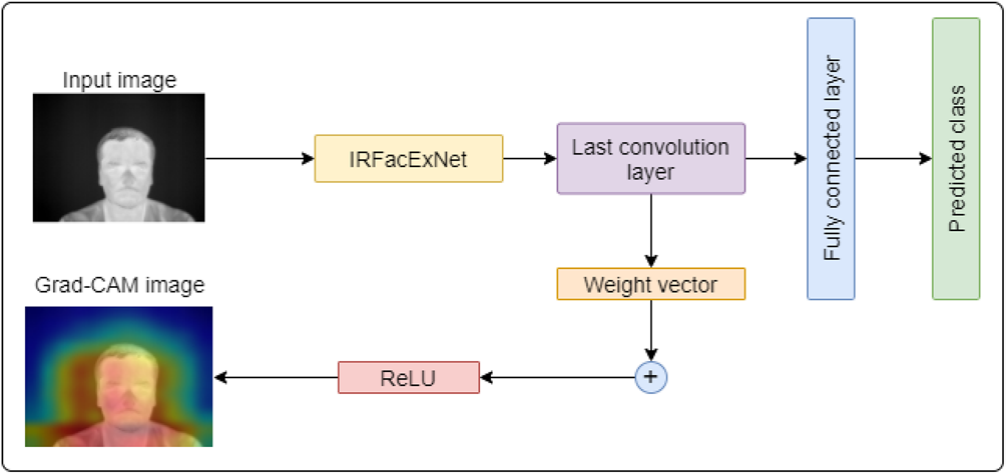
Outcome of the Grad-CAM analysis on an IR image for the proposed model—IRFacExNet. Image is developed using Draw.io^32^.

In this section, we have also tried to interpret the working procedure of the model used for the facial expressions classification. The interpretation of the model is required as facial expressions depend on different combinations of facial landmarks like eyebrows, nose, and lips, etc. Interpretation helps to verify the model whether it is detecting the correct areas of an image before making a decision. Selvaraju et al.^39^ have introduced a Gradient Weighted Class Activation Mapping (Grad-CAM) technique that helps to visualize the explainability of any deep learning model. Figure 10 pictorially demonstrates the functionality of Grad-CAM applied in our model. The process takes an image as input. The detection technique is applied to the image using the proposed model. After the successful calculation of the predicted class, Grad-CAM is applied to any of the convolution layers. In our case, we have considered the last layer for Grad-CAM analysis.

The gradient information flow the last convolution layer of the proposed deep learning model is used by Grad-CAM. The information is useful to interpret the decision of each neuron. This in turn helps to interpret the whole deep learning model. To calculate the class discriminative localization map of width *w* and height *h* for any class *c*, we first compute the output probability, $y^c$, of the class *c*. This probability is calculated before the softmax function. Then the gradient of $y^c$ is calculated with respect to $A^k$, the feature maps of a convolution layer. These gradients are then global average-pooled to obtain the neuron importance weights $\alpha ^k$ for the target class, as shown in Eq. (2).

2 $$\begin{aligned} \alpha ^c_k = \frac{1}{Z}\sum \limits _i \sum \limits _j \left( \frac{\partial y^c}{\partial A^k_{ij}} \right) \end{aligned}$$

After calculating $\alpha _k$ of the target class *c*, a weighted combination of activation maps is performed. Then it is followed by an activation function. The activation function used is ReLU (Rectified linear unit) as shown in Eq. (3). ReLU is applied because we are only interested in the visualization of the features having a positive influence on the class of interest. If ReLU was not used, then other features would have been considered for visualization contributing negatively to the prediction of the class. This results in a coarse heatmap of the same size as that of the convolutional feature maps. We have applied ReLU to the linear combination because we are only interested in the features that have a positive influence on the class of interest. Without ReLU, the class activation map highlights more than it requires and hence achieves low localization performance.

3 $$\begin{aligned} Grad{-}CAM_c=ReLU\left( \sum \limits _k \alpha ^c_k A^k \right) \end{aligned}$$

**Figure 11:**
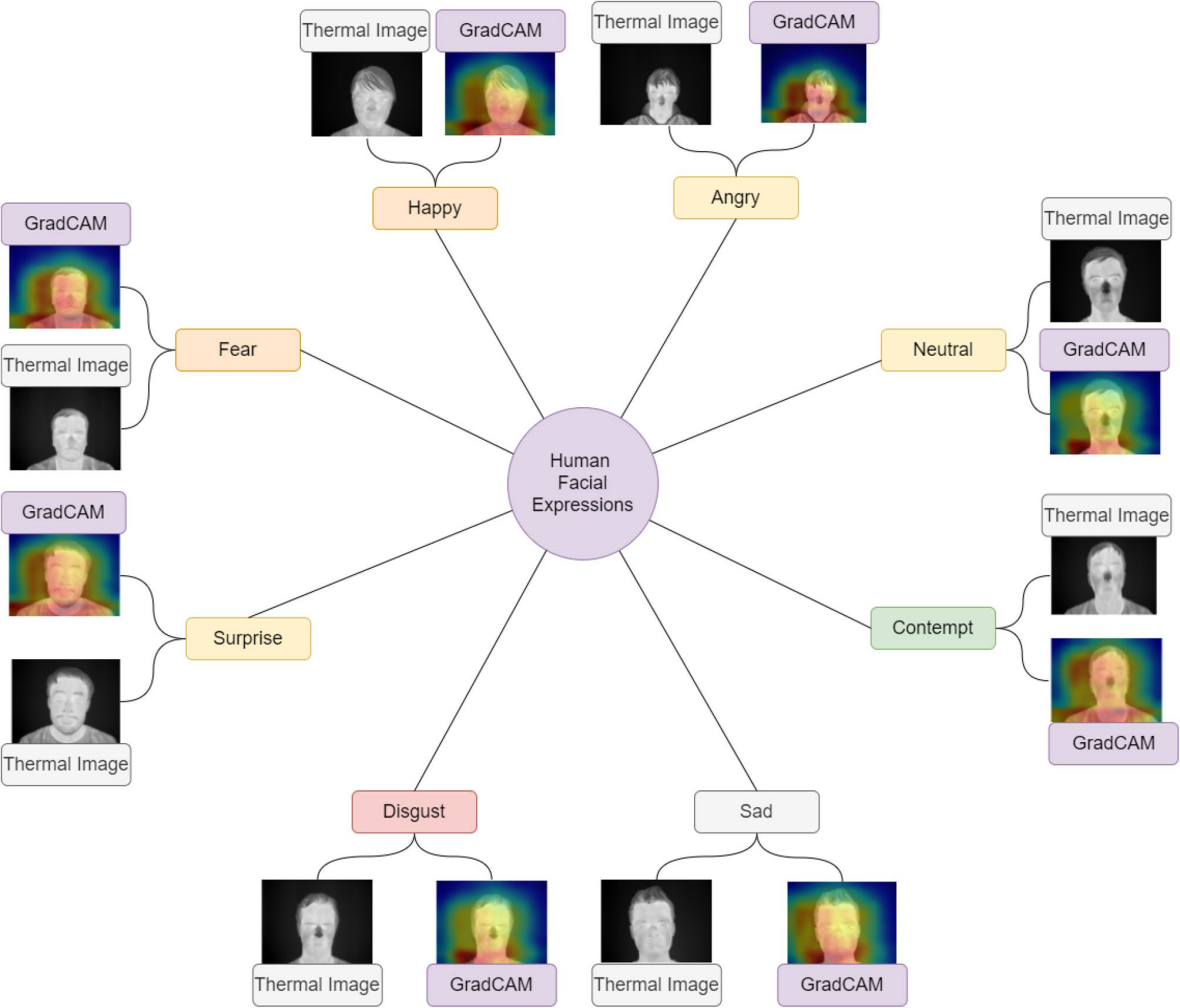
Grad-CAM output on IRFacExNet for IR images having different expressions. Image is developed using Draw.io^32^.

Figure 11 illustrates Grad-CAM output of different expressions of IR facial images.

**Figure 12:**
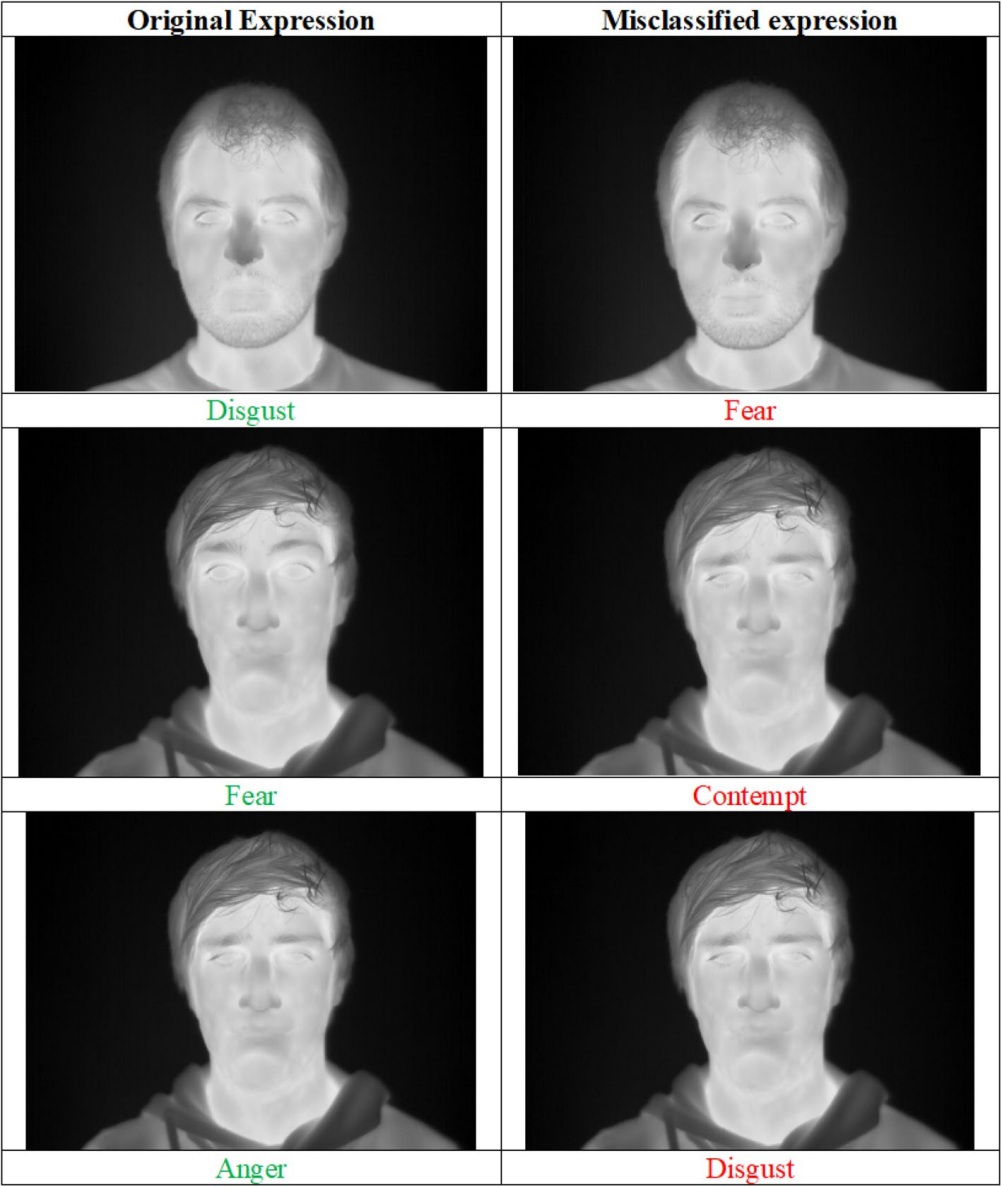
Some confusing facial expressions. The original expressions are marked in green color and the corresponding misclassified pair is marked in red. Image is developed using Draw.io^32^.

It can be observed that for each facial expression, the Grad-CAM output highlights the facial structure most prominently. This visualization confirms that the feature extracted by IRFacExNet are prominent and focuses on various facial landmarks. Then using the combination, it classifies the facial expressions. In the expression of fear, the two eye-brows can be seen blue-colored. But the space around the face is blue. This means that it uses the features of eyebrows that are most important. Similarly, for the disgust expression, the whole facial landmarks including nose, eye-brows, and mouth have been used as the most important features to classify the expression as disgust (Fig. 12).

**Figure 13:**
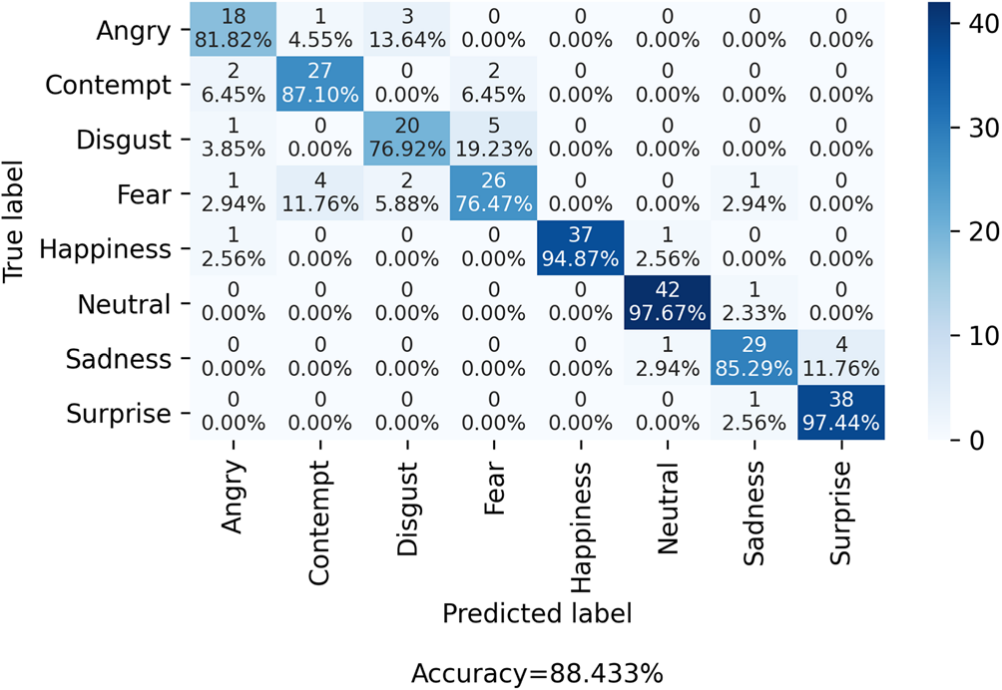
Confusion matrix of the IRFacExNet model.

From the confusion matrix shown in Fig. 13, it can be observed that the model gets confused for a similar type of expressions a human makes while feeling different emotions. For example, the highest misclassification has been observed between the expressions Disgust and Fear, which are strongly correlated to each other. These expressions are really confusing to create a distinction between them. The second highest wrong estimation has been found for the expressions between Anger and Disgust. An interesting phenomenon can be observed that expression of Fear has the lowest recognition rate of 76.47% and is mostly detected as the expression of Anger, Contempt, Disgust, and Sadness. Figure 12 highlights the most confusing pairs of facial expressions owing to which they get misclassified.

As discussed above, many times, humans make indistinguishable faces during expressing these emotions, and this causes the model to mislead. Also, it is to be noted here that the dataset we consider here for evaluation is comparatively a new one, and hence we have not found many works which also considered this dataset. However, there is still a lot of improvement in the recognition rate compared to the classification rate reported by Kopaczka et al.^12^. Table 4 compares the efficiency of the proposed method with some previously developed methods for the considered database^12^. It can be seen that our technique outperforms the previous recognition systems by achieving 88.433% classification accuracy. The key reasons for higher accuracy are:

- The proposed IRFacExNet model is very deep which helps to extract the dominanat features from the input images leading to better classification accuracy.
- Due to the presence of depthwise convolution operations in model, it extracts features over a wider area.
- The inclusion of snapshot ensemble strategy helps to improve the performance and robustness of the model.
- Besides, the IRDatabase used is fairly balanced. It ensures that the model is not biased towards a particular class.

**Figure 14:**
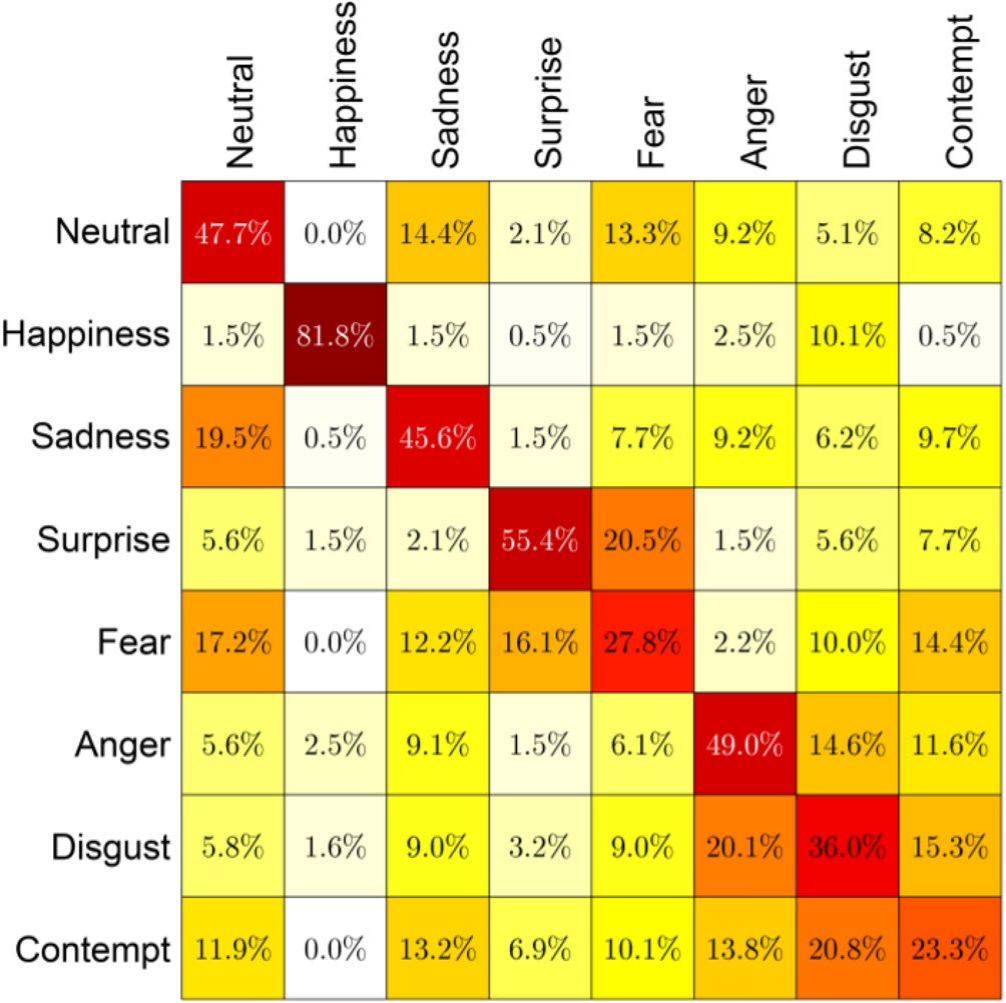
Confusion matrix of the compared method presented by Kopaczka et al.^12^.

The obtained confusion matrix from the proposed methodology when compared with confusion matrix (Fig. 14 ) obtained from Kopaczka et al.^12^ indicates the improvements in recognition accuracy in both class-wise and overall. This rise in recognition accuracy is the result of using DL methods. In the case of Neutral and surprise expressions, the precision is improved by almost a factor of 2. The lowest recognition accuracy reported was for expression of contempt and in our case, it is for Fear. These two expressions are highly correlated due to which two input samples of contempt are misclassified as fear in our study. Due to the lack of benchmark results on the employed database using DL methods, we have implemented the technique proposed in^28^ on the considered database which produces an accuracy of 79.54%.

**Table 4:** Performance comparison of the proposed FER method with some past methods evaluated on IRDatabase.

Work references	Method	Accuracy (%)
Kopaczka et al.^12^	HOG-SVM	46.7
Kopaczka et al.^40^	HOG features with Random Forest classifier (Neutral, Happy, Sad and Surprised)	65.7
Prabhakaran et al.^28^	ResNet152-V2	79.54
Proposed method	***IRFacExNet***	**88.433**

Bold value indicates result of the proposed methodology.

## Additional experiments

To establish the generalizability of the proposed approach, we have conducted a similar experiment on another database called Tufts Face Database^41^. The database includes over 10,000 images from 113 individuals from more than 15 different countries, belonging to different genders, ages, and ethnic backgrounds. We have considered only the infrared thermal images for thermal FER purposes. This dataset contains a total of 565 images from 113 participants with 5 images per participant for each expression. Images of this database are of five types—(a) neutral expression, (b) smile, (c) eyes close (sleepy), (d) exaggerated shocked expression, and (e) with sunglasses. We have maintained all the previously used hyperparameters and ran our experiment on this database. The obtained accuracy is 97.06%. The result so obtained is competitive when compared to existing study like TERNet^42^ which reported an accuracy of 96.2%. Kamath et al.^42^ developed the TERNet model for the FER corresponding to thermal images. They eliminated the last class corresponding to images with sunglasses. Several kinds of preprocessing techniques were utilized like adaptive histogram equalization, guided filtering, and intensity adjustment techniques. Along with these, they employed data augmentation to improve the model’s performance. On the other hand, we have not utilized any kind of image processing techniques or synthetic data to get better results. The IRFacExNet model itself extracts distinctive features using the Residual and Transformation units, and provides satisfactory results. This proves the robustness of the proposed approach. The summary of results on the Tufts database can be found in Table 5.

**Table 5:** Summary of results for Tufts face database.

Work references	Method used	Accuracy (%)
Kamath et al.^42^	VGG-16	93.4
	ResNet-18	94.2
	VGG-19	94.8
	VGG- Face	96.2
**Proposed method**	**IRFacExNet model**	**97.06**

Bold value indicates name and accuracy of the proposed methodology.

## Conclusion

HFER is an active research area in the domain of computer vision. In this paper, we have proposed an efficient deep learning model, called *IRFacExNet* for the recognition of human expressions from thermal images. Going by the current research trend, to develop this model, we have relied on a DCNN architecture. We have used two structural units namely, Residual Unit and Transformation Unit having their distinct strengths and these are able to extract useful features from the human faces used for the detection of the various expressions. This naïve model is able to achieve a recognition rate of 82.836%. To make the recognition system more robust, we have employed the Snapshot ensemble technique that has been trained using a cyclic learning rate scheduler. The predictions of the multiple models obtained after the Snapshot ensemble are combined to achieve a better prediction model. The ensemble system is able to achieve a state-of-the-art accuracy of 88.433% on the considered database. Although the proposed ensemble model produces a good recognition accuracy, however, there are some scopes for further improvement.The training of IRFacExNet model is computationally expensive compared to the naive model. The long training time can be reduced by the model’s architectural optimization which may reduce its complexity. Apart from that some attention mechanism in the CNN architecture can be added which might help to reduce the misclassification among similar types of expressions. To enhance the robustness of the proposed model, the recognition framework can be aided with multimodal sensors that may help to find facial features^43,44^. Again, towards making the model more explainable several strategies can be adopted to decipher the decision-making process of deep learning models^45^. It might help to make high-level decisions and be confident as the decisions are driven by combinations of data features specifically selected to achieve the desired results. To obtain the most relevant features, some feature selection algorithms can also be employed that might decrease the computational overhead. Apart from that the proposed model can be evaluated on other large-sized databases which would help us to ensure the robustness of the proposed model.
